# Use of a T cell interferon gamma release assay in the investigation for suspected active tuberculosis in a low prevalence area

**DOI:** 10.1186/1471-2334-9-105

**Published:** 2009-07-03

**Authors:** Niclas Winqvist, Per Björkman, Ann Norén, Håkan Miörner

**Affiliations:** 1 Infectious Disease Research Unit, Department of Clinical Sciences, Lund University, Malmö University Hospital, Malmö, Sweden; 2 Regional department of Infectious Disease Control and prevention, Malmö, Sweden; 3 Medical microbiology, Department of laboratory medicine, Lund University, Lund, Sweden

## Abstract

**Background:**

In settings with low background prevalence of tuberculosis (TB) infection, interferon-γ release assays (IGRA) could be useful for diagnosing active TB. This study aims to evaluate the performance of QuantiFERON^®^-TB Gold (QFT-G) in the investigation for suspected active TB, with particular attention to patients originating in high-incidence countries. Furthermore, factors associated with QFT-G results in patients with active TB were assessed.

**Methods:**

From patients investigated for clinically suspected active TB, blood was obtained for QFT-G testing, in addition to routine investigations. Positive (PPV) and negative (NPV) predictive values for QFT-G were calculated, comparing patients with confirmed TB and those with other final diagnoses. QFT-G results in TB patients originating from countries with intermediate or high TB incidence were compared with QFT-G results from a control group of recently arrived asymptomatic immigrants from high-incidence countries. Factors associated with QFT-G outcome in patients with confirmed TB were assessed.

**Results:**

Among 141 patients, 41/70 (58.6%) with confirmed TB had a positive QFT-G test, compared to 16/71 (22.6%) patients with other final diagnoses, resulting in overall PPV of 71.9% and NPV of 67.6%. For patients with pulmonary disease, PPV and NPV were 61.1% and 67.7%, respectively, and 90.5% and 66.7% for subjects with extrapulmonary manifestations. Comparing patients from high-incidence countries with controls yielded a PPV for active TB of 76.7%, and a NPV of 82.7%. Patients with confirmed TB and positive QFT-G results were characterized by a lower median peripheral white blood cell count (5.9 × 10^9^/L vs. 8.8 × 10^9^/L; *P *< 0.001) and a higher median body mass index (22.7 vs. 20.7; *P *= 0.043) as compared to QFT-G-negative TB patients.

**Conclusion:**

The overall PPV and NPV of QFT-G for identifying active TB were unsatisfactory, especially for pulmonary disease. Thus, the usefulness of QFT-G for this purpose is questionable. However, a high PPV was observed for extrapulmonary TB and QFT-G might be considered in the diagnostic process in this situation. The PPV and NPV for identifying active TB among persons originating from regions with high-and intermediate TB incidence was similar to that observed in subjects originating in the low-incidence region.

## Background

Even in low prevalence regions, TB is a common differential diagnosis in infectious disease practice, and different manifestations of TB may be encountered in all medical disciplines. Whereas most cases of pulmonary tuberculosis can be easily identified by detection of bacteria in airway secretions, the diagnosis of extrapulmonary TB is often difficult to establish. Recently, new diagnostic kits for tuberculosis infection, known as interferon-gamma release assays (IGRA:s), have become commercially available. T-SPOT^®^. TB (Oxford Immunotec Ltd.) is an assay that enumerates TB specific T-lymphocytes by Elispot technique [1], whereas QuantiFERON^®^-TB and QuantiFERON^®^-TB Gold (QFT-G; Cellestis Ltd.), are based on ELISA-techniques that measure γ-interferon released by TB specific T-lymphocytes [2]. Both of these tests appear to have higher specificity than the tuberculin skin test (TST) for TB infection [3-6], but the absence of a definite method to diagnose latent TB infection makes direct comparison between diagnostic methods impossible. In low-prevalence regions, TST is of limited value for detection of TB infection, since a high proportion of reactive skin tests have other aetiologies than TB [7], such as recent BCG vaccination or exposure to environmental mycobacteria. In addition to low specificity, the use of TST as a screening tool in patients with suspected active TB is hampered by high rates of false negative reactions in patients with active TB, especially in case of underlying immunodeficiency [8,9]. In the near future IGRA:s may even replace TST as a test for diagnosing latent TB. However, at present their role in the diagnostic process for suspected active infection remains uncertain.

Like TST, IGRA:s cannot differentiate between active and latent TB. A number of studies examining IGRA results in peripheral blood from patients with active TB has been published, and have found surprisingly low sensitivity [10-13]. Thus, their role in diagnosing active TB remains uncertain. However, promising results have recently been published with IGRA testing performed on mononuclear cells obtained from the site of infection e.g. from broncho-alveolar lavage [14].

Sweden has a low incidence of TB (5.5 per 100 000 inhabitants in 2007) [15]. In this setting with a low background prevalence of latent TB, IGRA tests could be useful in the initial diagnostic process in patients with suspected TB [16]. During the last decades, clusters of TB have been observed in large urban centres [16,17]. Considering the epidemiological situation in many industrialized countries with a disproportionate fraction of TB cases occurring in immigrants from high-incidence countries, the ability of new diagnostic methods to detect patients with active or latent TB in immigrant populations is of great importance.

The principal objective of this study was to define the usefulness of QFT-G in the diagnostic procedure for suspected active tuberculosis among patients in Southern Sweden by calculating PPV and NPV for QFT-G. In order to assess the performance among persons originating from high-prevalence areas, a group of healthy recently arrived immigrants from such countries were compared to those in patients with similar origin. We also aimed to identify factors associated with QFT-G results in patients with confirmed active TB.

## Methods

### Study population

This study was conducted in Malmö and Lund in Southern Sweden (population ca 400 000). In this area, about 40 cases of TB are reported annually. Study subjects belonged to two categories: case patients with suspected tuberculosis, and healthy immigrants who had recently arrived in Sweden from countries with a high prevalence of TB.

Patients were recruited consecutively from the departments of infectious and pulmonary diseases in the university hospitals in Malmö and Lund between September 2004 and February 2007. Inclusion criteria were: age over 15 years, written informed consent, and ongoing investigation for symptoms or signs for which TB was considered as a differential diagnosis. Medical investigation typically included microbiological testing from affected organs, radiological investigations and histopathological studies, according to disease manifestations. Patients were excluded if TB treatment had been initiated more than 1 week prior to study entry, or if TB investigations were done as part of a routine diagnostic package with no definite clinical suspicion of TB (e.g. patients undergoing bronchoscopy for investigation of pulmonary lesions, in which TB cultures are routinely obtained). For patients with suspected TB, an interview and record-based questionnaire covering general medical history, risk factors for exposure to TB and details concerning ongoing disease and results of medical investigations was completed by either a physician or a specialized nurse. TST was recommended, but not required, for study inclusion.

Since around 80 percent of all TB cases in the study area occur among immigrants from countries with high or intermediate TB incidence [18] we chose to compare results in immigrant patients with active TB with those in healthy subjects with similar geographical origin. A control group of recently (within 7 months) arrived immigrants, aged over 15 years and without significant medical history, were recruited from a primary care centre performing health examinations, which are offered to all immigrants arriving in Sweden. Recruitment of immigrants was performed between March 2006 and September 2006. These examinations include a general health questionnaire, voluntary HIV test, chest X-ray and TST.

A TB case was defined as an individual with symptoms and signs compatible with TB and isolation of, or detection of nucleic acid from, *Mycobacterium tuberculosis *complex from a clinical specimen (microbiologically confirmed TB), or as a patient with tuberculosis clinically suspected by an expert physician, and responding to anti-tuberculosis treatment[19].

### QuantiFERON-TB^® ^Gold test

Whole blood was collected in heparin-containing tubes for the QuantiFERON^®^-TB Gold test (Cellestis Ltd, Victoria, Australia). Samples were transported to the laboratory within 4 hours and incubation with antigens was initiated within 7 hours after collection. Antigens provided in the QFT-G test kit were the 6 kDa early secreted antigenic target (ESAT-6) and culture filtrate protein 10 (CFP-10); in addition to these, TB7.7 (kindly provided by Karin Weldingh; Statens Serum Institut, Copenhagen, Denmark) and purified protein derivative (PPD; Art. No 2390; Statens Serum Institut) were used for incubation experiments. After 16 – 24 hours of incubation at 37°C in 5% CO_2_, supernatants were collected and frozen at -20°C until analysis. Concentrations of interferon-gamma (INF-γ) in 50 μL of plasma from whole-blood stimulated with the antigens, mitogen (positive control) or saline (negative control) were measured using the ELISA kit provided with the QFT-G package. A positive result was defined as a concentration of INF-γ of ≥0.35 IU/mL after subtraction of the INF-γ concentration from the negative control in at least 1 of the specific antigens ESAT-6, CFP-10 or TB7.7. The INF-γ concentration in the antigen stimulated wells had to be at least half of the concentration of the negative control for the result to be valid. If the mitogen response minus the negative control was < 0.5 the result was defined as indeterminate.

The results of the QFT-G tests were not available to the physicians in charge of the patients, and thus did not influence the diagnostic procedure.

### Tuberculin skin test

TST was done by intradermal injection of 0.1 ml (2 TU) PPD (RT23; Statens Serum Institut, Copenhagen, Denmark). Skin induration was measured after 72 hours and was interpreted according to the recommendations by the manufacturer. In most cases TST was done prior to QFT-G or at the same time.

### Statistical methods

Positive (PPV) and negative (NPV) predictive values of the QFT-G test among patients were calculated using a final TB diagnosis as true positive and other final diagnoses as true negative outcome. To facilitate comparisons with other studies, calculations of sensitivity and specificity for the diagnosis of active TB were also performed. McNemar test was used to test for overall accuracy. Patients with indeterminate QFT-G results were excluded from the analysis.

Patients originating from high and intermediate prevalence regions were compared with healthy immigrant controls to determine PPV and NPV for active TB of both QFT-G and TST in the immigrant population. In patients with confirmed TB, factors associated with positive or negative QFT-G result were first determined by univariate regression analysis. In order to control for possible confounding factors, variables were then put into a multiple regression model.

For dichotomous variables an odds ratio was calculated. Differences in odds ratio with a p-value <.05 was considered significant. Odds ratios were defined as the exponent of the estimated beta for dichotomous variables. When comparing means for continuous variables the Student's t-test was used for variables with normal distribution. For other variables the Mann-Whitney's non-parametric test was used.

The study procedure was approved by the ethical vetting board, Lund University, Sweden.

## Results

### Patients with suspected tuberculosis

In 70 of 141 patients, the diagnosis of TB was established, whereas 71 patients received another final diagnosis (most frequent were bacterial pneumonia [17], cancer [5], and other respiratory diseases [4]). Characteristics of the patients are shown in Table 1. TB was bacteriologically confirmed either by culture, microscopy or polymerase chain reaction (PCR) in 61 patients (87%); in the remaining 9 patients, diagnosis was based on clinical criteria, supported by radiological and/or histopathological findings, and response to anti-tuberculosis therapy.

**Table 1:** Characteristics of patients investigated for suspected active tuberculosis, and controls

	Tuberculosis n = 70	Other final diagnoses n = 71	Controls n = 98
Women (%)	40 (57.1)	35 (49.3)	35 (35.7)
Median age (years)	38	58	32
Median BMI	22	23	
Origin from country with high or intermediate TB-incidence* (%)	52 (74.3)	23 (32.4)	98 (100)
Immune supression** (%)	19 (27.1)	28 (39.4)	0 (0.0)
Affected organ			
Lung (%)	44 (62.9)	60 (84.5)	
Pleura (%)	3 (4.3)	1 (1.4)	
Mediastinal lymph node (%)	1 (1.4)	2 (2.8)	
Peripheral lymph node (%)	10 (14.3)	1 (1.4)	
Joint/skeleto-muscular (%)	3 (4.3)	3 (4.3)	
Skin/soft tissue (%)	2 (2.8)	1 (1.4)	
Gastrointestinal/abdominal (%)	4 (5.7)	0 (0.0)	
Urogenital (%)	0 (0.0)	1 (1.4)	
Multiple locations (%)	3 (4.3)	2 (2.8)	

TB: Tuberculosis; BMI: Body Mass Index

* Defined as ≥20 cases/100 000 inhabitants

** HIV-infection (n = 6), immunosuppressive treatment (n = 18), diabetes (n = 9), alcohol or drug abuse (n = 9), cancer (n = 4), Mb Down (n = 1)

Patients originating from countries with an estimated TB-incidence of more than 20 new cases per 100 000 inhabitants according to the World Health Organization (WHO)[20] were significantly more likely to get a TB diagnosis compared to those from low incidence countries (52/75 vs. 18/66; *P *< 0.001). Patients with TB were also younger than those with another final diagnosis (median age 38 years vs. 58; *P *< 0.001). Other parameters analyzed did not differ significantly between patients with confirmed TB and non-TB cases.

### QuantiFERON-TB^® ^Gold results in patients with suspected tuberculosis

Results of QFT-G for respective patient categories are presented in Table 2. Ten subjects had indeterminate QFT-G results; these patients did not differ significantly from those with a positive or negative QFT-G result with regard to any of the parameters tested.

**Table 2:** Positive and negative predictive values of QuantiFERON^®^-TB Gold results in patients with confirmed TB and patients with other final diagnoses.

	Tuberculosis* n = 70	Other final diagnosis* n = 71	Positive predictive value	Negative predictive value
**All patients; n = 141**				
QFT-G positive	41	16		
QFT-G negative	24	50	71.9%	67.6%
QFT-G indeterminate	5	5		
**Suspected pulmonary TB; n = 104**				
QFT-G positive	22	14		
QFT-G negative	20	42	61.1%	67.7%
QFT-G indeterminate	2	4		
**Suspected extrapulmonary TB; n = 37**				
QFT-G positive	19	2		
QFT-G negative	4	8	90.5%	66.7%
QFT-G indeterminate	3	1		

QFT-G: QuantiFERON^®^-TB Gold; TST: Tuberculin skin test

* Bacteriological and/or clinical diagnosis

For the whole group of patients, the PPV and NPV of QFT-G was 71.9% and 67.6%, respectively. The sensitivity and specificity for QFT-G was 63.1% and 75.8% respectively (McNemar test for overall accuracy: *P *= 0.268).

Among 104 patients with pulmonary disease manifestations, QFT-G was positive in 22 of 44 TB-cases and in 14 out of 60 non-TB patients, (PPV 61.1%, NPV 67.7%; Table 2). In this subgroup the sensitivity was 52.4% and the specificity 75.0% (McNemar test: *P *= 0.392).

Thirty-seven patients had suspected extrapulmonary TB. Out of these, 21 had a positive QFT-G test, 12 had a negative test and in 4 subjects the QFT-G result was indeterminate (Table 2). Two of the 21 QFT-G-positive subjects with suspected extrapulmonary TB received a final diagnosis other than TB. The PPV and NPV of QFT-G in subjects with suspected extrapulmonary TB was 90.5% and 66.7% and sensitivity and specificity was 82.6% and 80.0% respectively (McNemar test: *P *= 0.687).

In the subgroup of patients originating from countries with high or intermediate TB incidence, PPV and NPV were 78.6% and 46.7% respectively (pulmonary and extrapulmonary disease manifestations combined).

### Immigrants from high and intermediate prevalence countries – comparison of QFT-G results among patients with tuberculosis and healthy controls

The TB incidence in the country of origin was lower among the 98 immigrant controls than for the 52 patients who originated from high and intermediate prevalence regions (mean 63 vs. 158 new cases/100 000 inhabitants; *P *< 0.001). The mean age of the control subjects was lower than that of the immigrant patients (31.8 years vs. 42.6; *P *< 0.001) and the male/female ratio was higher (63/35 vs. 41/42). The most common countries of origin were Iraq (58), the Palestine (11) and Somalia (7) for the immigrant controls, and Serbia (9), Somalia (9), Bosnia-Herzegovina (8), Iraq (8) and Afghanistan (8) for the immigrant patients.

Among the 52 immigrant patients with confirmed TB, 33 (63.5%) had positive QFT-G results. QFT-G results could be determined in 96 of 98 controls. Among these, 10 (10.4%) had a positive result. In control subjects with TST results, the induration was ≥10 mm in 39 of 85 (45.9%) persons.

When comparing subjects from high-prevalence countries, the PPV for a positive QFT-G in patients with active TB was 76.7%, and the NPV was 82.7%. The corresponding PPV and NPV for a TST with cut off of 10 mm was 44.3% and 93.9% respectively (Table 3). In terms of sensitivity and specificity the results were for QFT-G 67.3% and 89.6% respectively (McNemar test for overall accuracy: *P *= 0.327) and for TST 91.2% and 54.1% respectively (McNemar test: *P *< 0.001).

**Table 3:** Comparison of QFT-G and TST results in TB patients and controls with origin from country with intermediate or high TB incidence (n = 150).

	TB-patients* n = 52	Controls n = 98	Positive predictive value	Negative predictive value
QFT-G positive	33	10		
QFT-G negative	16	86	76.7%	84.3%
QFT-G indeterminate	3	2		
				
TST ≥ 10 mm	31	39		
TST <10 mm	3	46	44.3%	93.9%
TST not performed	18	13		

QFT-G: QuantiFERON^®^-TB Gold; TST: Tuberculin skin test

* Bacteriological and/or clinical diagnosis

### Factors associated with QFT-G result in patients with active tuberculosis

To further explore factors that may influence the QFT-G results in TB-patients, various parameters were tested. The findings are divided with regard to the variable's quality (dichotomous or scale) and are presented in Table 4 and 5. Positive QFT-G results were more common in patients with extrapulmonary TB than in those with pulmonary disease. The concentration of INF-γ was also significantly higher in patients with extrapulmonary symptoms compared to patients with pulmonary symptoms (mean 11.3 IU/mL vs. 1.9 IU/mL; Student's t-test: *P *< 0.001). The difference between women and men was borderline significant, with a higher likelihood of positive results in women (OR 2.70; 95% CI: 0.96–7.62; *P *= 0.0504).

**Table 4:** Dichotomous variables; association with QFT-G results in tuberculosis patients

	QFT positive	QFT negative	Odds ratio	95% CI interval	p-value
Male	14	14	2.70	0.96 – 7.62	0.0504
Female	27	10			
Origin from high or intermediate TB incidence country*	33	16	2.04	0.63 – 6.66	0.1710
Origin from low incidence country	8	8			
Born abroad and lived <5 years in Sweden	14	5	1.95	0.61 – 6.96	0.1970
Born in, or lived >5 years in, Sweden	27	19			
Extra pulmonary tuberculosis	19	4	4.31	1.25 – 14.87	0.0142
Pulmonary tuberculosis	22	20			
Sputum smear negative**	9	6	1.62	0.38 – 7.15	0.4666
Sputum smear positive	13	14			
Immunosuppressed patients ***	9	8	1.76	0.55 – 5.58	0.2357
Immune-competent patients	32	16			
TST ≥ 10 mm	27	9	3.00	0.37 – 24.50	0.3000
TST < 10 mm	2	2			

QFT: QuantiFERON^®^-TB Gold; CI: Confidence Interval; BMI: Body Mass Index (kg/m^2^); TST: Tuberculin skin test

5 patients with indeterminate results excluded

* High or moderate TB-incidence is defined as ≥20 cases/100 000 population

** For pulmonary TB only (result missing in 2 patients)

*** Immune suppression defined as HIV-infection, ongoing immunosuppressive treatment, cancer, diabetes, alcohol or drug abuse

**Table 5:** Means of scale variables and their association with QFT result in TB patients

	Number of observations	QFT positive	QFT negative	95% CI of difference	t-test	p-value
Age (years)	65	42	44	-12.4–7.9	-0.45	0.657
TB incidence of native country (new cases/100 000 pop.)	65	133	87	-17.7–110.0	1.45	0.157
Body Mass Index (BMI)	58	22.7	20.7	0.1–4.0	2.08	0.043*
Hemoglobin (Hb; g/L)	59	125	121	-0.7–14.3	1.01	0.315
ESR (mm)	41	45	56	-31.5–9.2	-1.12	0.272
C-reactive protein (CRP; mg/L)	59	42	79	-76.9–2.4	-1.91	0.065
Total leukocyte count (cells × 10^9^/L)	60	5.9	8.8	-4.3–-1.4	-4.02	<0.001*
TST (mm)	40	23	15	1.2–15.4	2.40	0.025*

* Significant difference of means

Mean score of body mass index (BMI; kg/m^2^) for QFT-G positive TB patients was 22.7 compared to 20.7 for QFT-G negative subjects (Student's t-test: *P *= 0.043). Patients with a positive QFT-G test had a significantly lower peripheral white blood cell count (WBC) than QFT-G negative subjects (mean 5.9 vs. 8.8 × 10^9^/L; Student's t-test: *P *< 0.001). In patients with TST results, the skin induration was significantly larger in QFT-G positive individuals (mean 23 mm and 15 mm respectively; Student's t-test: *P *= 0.025).

In order to account for confounding factors, parameters that may influence the QFT-G result were entered into a logistic regression model (Table 6). After controlling for sex and a diagnosis of extrapulmonary TB, the only significant difference was a lower WBC in QFT-G positive individuals.

**Table 6:** Multiple logistic regression analysis of factors potentially associated with QFT-results in TB-patients (after controlling for sex and extrapulmonary TB)

Factor	Number of observations	Est. beta-coefficient	OR*	95% CI	p-value
Bacteriologically verified diagnosis	65	-0.022	0.98	0.20–4.78	0.977
TST cut off 10 mm	40	-0.666	0.51	0.21–1.28	0.153
TST cut off 15 mm	40	1.384	3.99	0.72–22.15	0.114
Immune suppression**	65	-0.680	0.51	0.15–1.76	0.284
TB incidence of native country (new cases/100 000 pop.)	65	0.003			0.200
Body Mass Index (BMI)	58	0.146			0.110
Hemoglobin (mg/L)	59	0.022			0.181
Erythrocyte Sedimentation Rate (mm)	41	-0.005			0.995
C-reactive protein (CRP;mg/L)	59	-0.007			0.124
White blood cell count (WBC; × 10^9^/L)	60	-0.568			<0.001

*Odds ratios (defined as the exponent of the estimated beta) are not shown for TB incidence of native country, BMI, Hb, ESR, CRP and WBC since these variables are not dichotomous.

** Immune suppression defined as HIV-infection, ongoing immunosuppressive treatment, cancer, diabetes, alcohol or drug abuse

### Antigens for incubation experiments

The antigens added for incubation experiments, TB7.7 and PPD, did not show a significant effect on the results. Six positive QFT-G tests based on TB7.7 only were noted, 2 each in the TB patient group, the non-TB patient group and the group of healthy immigrants. QFT-PPD was used for experiments only and did not give rise to positive QFT-G results alone. The correlation between QFT-PPD and TST was 0.232 (Pearson's correlation test; *P *= 0.075). In TB patients there was a significant, negative correlation between WBC and QFT-PPD (-3.178; Pearson's correlation test; *P *= 0.003).

## Discussion

Correct diagnosis of tuberculosis remains a challenge in clinical practice, especially for extrapulmonary disease manifestations for which invasive procedures are required to detect mycobacteria in affected tissues. In this study, performed among patients investigated for suspected TB in a low-prevalence region, the overall usefulness of QFT-G as part of the initial diagnostic procedure was limited, with a low negative predictive value for identifying active TB. Interestingly, we observed that a negative QFT-G reaction was more common in pulmonary TB than among patients with extrapulmonary disease manifestations. Among patients with extrapulmonary disease, QFT-G performed much better. Most patients with extrapulmonary TB in our material had isolated peripheral lymphadenitis. Lymph node biopsies were obtained in several of these patients, and showed pronounced granulomatous inflammatory reaction; in addition, the mycobacterial load was low, since bacterial growth was only detected from biopsies and not in fine-needle aspirates. Such a presentation is common in peripheral tuberculous lymphadenitis in HIV-negative patients, and is related to a strong immune response [21], further supported by the finding of high IFN-γ secretion in such patients[22,23].

Our results on the PPV and NPV of IGRA in active TB are in agreement with those reported by others [10,13,24,25]. The limited sensitivity of IGRA tests in active TB could be related to the secretion of IFN-γ suppressive factors in active TB [26], or to the immunological capacity of the host to contain mycobacterial replication [23]. IFN-γ secretion has been shown to be inversely correlated to disease severity in patients with TB [23,27,28]. Lower rates of IFN-γ secretion and higher frequencies of indeterminate IGRA results have also been observed in HIV-infected subjects [29-31]. We did not find any correlation with QFT-G results and the presence of immunosuppression in our patients; however, the proportion of HIV-infected subjects in our material was rather low. For T-SPOT^®^. TB, indeterminate results have been associated with delayed handling times [32], whereas an evaluation of cytokine techniques under different circumstances found that ELISA performance was not much affected by handling conditions [33]. The rate of indeterminate results was 7.1% (10/141) in this study, which is at the same level as reported in other studies on patients with suspected clinically active TB disease [34]. The rate of false negative was 34% in this study (24/70). There are large variations in the literature but several other studies have reported a similar low sensitivity in patients with active tuberculosis [10,13,35,36]. In our study, all samples were sent to the laboratory and processed within 7 hours; thus, it is unlikely that technical problems explain the high frequency of negative and indeterminate results in patients with confirmed active TB.

It has been suggested to lower the cut-off limits for the IGRA tests in low incidence settings in order to improve sensitivity[37]. Recalculating PPV and NPV in our material using the cut-off limits 0.20 IU/mL and 0.10 IU/mL reduced PPV for patients with pulmonary and extrapulmonary TB respectively. Only the lowest cut-off limit raised the NPV slightly (data not shown). Hence, lowering cut-off limits for the QFT-G would not benefit the test's performance in our setting.

The magnitude and characteristics of immune response are important factors for the pathogenesis and disease manifestations in tuberculosis, and they are likely to be associated with the rate of IFN-γ secretion. A parallel may be drawn to leprosy, another mycobacterial infection in which the immune response is critical for disease presentation. In an Indian study, significantly higher IFN-γ secretion was detected in patients with tuberculoid variants of leprosy, as opposed to lepromatous forms (in which the Th-1 response is weak) [38]. The association between WBC in peripheral blood and QFT-G outcome in our TB patients could be a reflection of the same phenomenon, and are in agreement with findings reported by Shang and co-workers, showing enhanced peripheral blood leukocytosis in mice with targeted mutation of chemokine receptor 1 and reduced IFN-γ secretion [39]. The lower BMI in QFT-G negative TB-patients is also likely to be related to a reduced capacity for INF-γ secretion in patients with advanced disease.

When assessing the usefulness of IGRA testing for detecting active TB, the immunological profile of the studied patients should thus be taken into account. Our results may not be applicable for HIV-positive patients. Furthermore, several IGRA techniques have been developed, and in a meta-analysis of commercially available IGRA:s differences with regard to sensitivity and applicability have been found [6,16]. We used an extended, in-house variant of QuantiFERON^®^-TB Gold, with the addition of another MTB specific antigen (TB7.7) apart from those included in the commercial kit. However, the inclusion of this antigen did not appear to affect test outcome. In this study, we have not compared the two different IGRA test methods. Although comparisons between QFT-G and T-SPOT^®^. TB suggest that the latter may be more sensitive for diagnosing latent TB, the significance of this difference in clinical practice is not clear. Furthermore, QFT has the advantage of being easier and less expensive to perform than T-SPOT^®^. TB testing. Both IGRA test methods have superior specificity for TB compared to TST, but sensitivity is probably lower (depending on the TST cut-off chosen to define TB infection). However, in analogy with QFT-G, false negative TST reactions are common in severe forms of TB [40].

An important finding from our study is that QFT-G had similar positive and negative predictive values for active TB among native Swedes and immigrants from high-prevalence countries – a group that presently constitutes the majority of TB patients in Sweden as well as in most other European countries. This is somewhat surprising in view of the limited sensitivity of QFT-G for active TB, and the presumed high prevalence of latent TB among the healthy immigrant controls (TST ≥ 10 mm in 39/85, 46%). With regard to the fact that the incidence of TB in the countries of origin among immigrant patients was higher than in controls, it may be anticipated that the exposure to TB was different between these groups. Still, this observation raises further questions regarding the significance of QFT-G and TST for identification of persons infected with *M. tuberculosis*.

## Conclusion

The overall usefulness of QFT-G for identifying active TB was limited among patients investigated for suspected TB in a typical setting in a North-European low-prevalence region. Poor performance was more common in patients with pulmonary disease, whereas the PPV for QFT-G was satisfactory among patients with extrapulmonary disease manifestations. Since TB is more difficult to diagnose in such cases, we suggest that it may be reasonable to consider QFT-G for screening patients with suspected extrapulmonary TB. Furthermore, the PPV and NPV of QFT-G for detection of actitve TB among immigrants from high and intermediate prevalence regions seem to be similar to that observed in non-immigrants.

## Competing interests

The authors declare that they have no competing interests.

## Authors' contributions

NW, PB and HM were responsible for planning the study, analyzing the results and drafting the manuscript. HM designed the antigen panel used for QFT-G testing. NW, AN and PB collected the study material and coordinated the study. All authors have read and approved the manuscript.

## Pre-publication history

The pre-publication history for this paper can be accessed here:

http://www.biomedcentral.com/1471-2334/9/105/prepub

## References

[B1] Lalvani A Pathan AA Durkan H Wilkinson KA Whelan A Deeks JJ Reece WH Latif M Pasvol G Hill AV Enhanced contact tracing and spatial tracking of Mycobacterium tuberculosis infection by enumeration of antigen-specific T cells Lancet 2001 357 9273 2017 2021 10.1016/S0140-6736(00)05115-1 11438135

[B2] Ravn P Munk ME Andersen AB Lundgren B Nielsen LN Lillebaek T Soerensen IJ Andersen P Weldingh K Reactivation of tuberculosis during immunosuppressive treatment in a patient with a positive QuantiFERON-RD1 test Scandinavian journal of infectious diseases 2004 36 6–7 499 501 10.1080/00365540410015222 15307581

[B3] Fietta A Meloni F Cascina A Morosini M Marena C Troupioti P Mangiarotti P Casali L Comparison of a whole-blood interferon-gamma assay and tuberculin skin testing in patients with active tuberculosis and individuals at high or low risk of Mycobacterium tuberculosis infection American journal of infection control 2003 31 6 347 353 10.1016/S0196-6553(02)48240-5 14608301

[B4] Meier T Eulenbruch HP Wrighton-Smith P Enders G Regnath T Sensitivity of a new commercial enzyme-linked immunospot assay (T SPOT-TB) for diagnosis of tuberculosis in clinical practice Eur J Clin Microbiol Infect Dis 2005 24 8 529 536 10.1007/s10096-005-1377-8 16133410

[B5] Mori T Sakatani M Yamagishi F Takashima T Kawabe Y Nagao K Shigeto E Harada N Mitarai S Okada M Specific detection of tuberculosis infection: an interferon-gamma-based assay using new antigens American journal of respiratory and critical care medicine 2004 170 1 59 64 10.1164/rccm.200402-179OC 15059788

[B6] Pai M Zwerling A Menzies D Systematic review: T-cell-based assays for the diagnosis of latent tuberculosis infection: an update Annals of internal medicine 2008 149 3 177 184 18593687 10.7326/0003-4819-149-3-200808050-00241 PMC2951987

[B7] Wang L Turner MO Elwood RK Schulzer M FitzGerald JM A meta-analysis of the effect of Bacille Calmette Guerin vaccination on tuberculin skin test measurements Thorax 2002 57 9 804 809 12200526 10.1136/thorax.57.9.804 PMC1746436

[B8] Bloom BR Small PM The evolving relation between humans and Mycobacterium tuberculosis The New England journal of medicine 1998 338 10 677 678 10.1056/NEJM199803053381008 9486998

[B9] Boussiotis VA Tsai EY Yunis EJ Thim S Delgado JC Dascher CC Berezovskaya A Rousset D Reynes JM Goldfeld AE IL-10-producing T cells suppress immune responses in anergic tuberculosis patients The Journal of clinical investigation 2000 105 9 1317 1325 10792007 10.1172/JCI9918 PMC315449

[B10] Ferrara G Losi M D'Amico R Roversi P Piro R Meacci M Meccugni B Dori IM Andreani A Bergamini BM Use in routine clinical practice of two commercial blood tests for diagnosis of infection with Mycobacterium tuberculosis: a prospective study Lancet 2006 367 9519 1328 1334 10.1016/S0140-6736(06)68579-6 16631911

[B11] Goletti D Carrara S Vincenti D Saltini C Rizzi EB Schinina V Ippolito G Amicosante M Girardi E Accuracy of an immune diagnostic assay based on RD1 selected epitopes for active tuberculosis in a clinical setting: a pilot study Clin Microbiol Infect 2006 12 6 544 550 10.1111/j.1469-0691.2006.01391.x 16700703

[B12] Lee JY Choi HJ Park IN Hong SB Oh YM Lim CM Lee SD Koh Y Kim WS Kim DS Comparison of two commercial interferon-gamma assays for diagnosing Mycobacterium tuberculosis infection Eur Respir J 2006 28 1 24 30 10.1183/09031936.06.00016906 16611658

[B13] Mazurek GH Weis SE Moonan PK Daley CL Bernardo J Lardizabal AA Reves RR Toney SR Daniels LJ LoBue PA Prospective comparison of the tuberculin skin test and 2 whole-blood interferon-gamma release assays in persons with suspected tuberculosis Clin Infect Dis 2007 45 7 837 845 10.1086/521107 17806047

[B14] Jafari C Lange C Suttons's Law: Local Immunodiagnosis of Tuberculosis Infection 2008 36 6 510 514 10.1007/s15010-008-8089-9 18931970

[B15] Tuberculosis in Sweden http://www.smittskyddsinstitutet.se/statistik/tuberkulos

[B16] Menzies D Pai M Comstock G Meta-analysis: new tests for the diagnosis of latent tuberculosis infection: areas of uncertainty and recommendations for research Annals of internal medicine 2007 146 5 340 354 17339619 10.7326/0003-4819-146-5-200703060-00006

[B17] Kan B Berggren I Ghebremichael S Bennet R Bruchfeld J Chryssanthou E Kallenius G Petersson R Petrini B Romanus V Extensive transmission of an isoniazid-resistant strain of Mycobacterium tuberculosis in Sweden Int J Tuberc Lung Dis 2008 12 2 199 204 18230254

[B18] Winqvist N Andersen PH Lillebaek T Bjorkman P Miorner H Demographics of tuberculosis in an emerging EU region in southern Scandinavia Scandinavian journal of infectious diseases 2006 38 11–12 1033 1039 10.1080/00365540600868313 17148073

[B19] Welfare TNBoHa Case definitions on report in accordance with the Swedish Disease Control and Prevention Act 2008 Welfare TNBoHa: The National Board of Health and Welfare

[B20] Global tuberculosis control – surveillance, planning, financing http://www.who.int/tb/publications/global_report/2008/en/

[B21] Polesky A Grove W Bhatia G Peripheral tuberculous lymphadenitis: epidemiology, diagnosis, treatment, and outcome Medicine 2005 84 6 350 362 10.1097/01.md.0000189090.52626.7a 16267410

[B22] Habte A Geletu M Olobo JO Kidane D Negesse Y Yassin MA Kifle B Abate G Harboe M Aseff A T cell mediated immune responses in patients with tuberculous lymphadenitis from Butajira, southern Ethiopia Ethiopian medical journal 2004 42 Suppl 1 29 35 16895017

[B23] Pathan AA Wilkinson KA Klenerman P McShane H Davidson RN Pasvol G Hill AV Lalvani A Direct ex vivo analysis of antigen-specific IFN-gamma-secreting CD4 T cells in Mycobacterium tuberculosis-infected individuals: associations with clinical disease state and effect of treatment J Immunol 2001 167 9 5217 5225 11673535 10.4049/jimmunol.167.9.5217

[B24] Dewan PK Grinsdale J Kawamura LM Low sensitivity of a whole-blood interferon-gamma release assay for detection of active tuberculosis Clin Infect Dis 2007 44 1 69 73 10.1086/509928 17143818

[B25] Pai M Joshi R Bandyopadhyay M Narang P Dogra S Taksande B Kalantri S Sensitivity of a whole-blood interferon-gamma assay among patients with pulmonary tuberculosis and variations in T-cell responses during anti-tuberculosis treatment Infection 2007 35 2 98 103 10.1007/s15010-007-6114-z 17401714 PMC2951985

[B26] Pai RK Convery M Hamilton TA Boom WH Harding CV Inhibition of IFN-gamma-induced class II transactivator expression by a 19-kDa lipoprotein from Mycobacterium tuberculosis: a potential mechanism for immune evasion J Immunol 2003 171 1 175 184 12816996 10.4049/jimmunol.171.1.175

[B27] Goletti D Butera O Bizzoni F Casetti R Girardi E Poccia F Region of difference 1 antigen-specific CD4+ memory T cells correlate with a favorable outcome of tuberculosis The Journal of infectious diseases 2006 194 7 984 992 10.1086/507427 16960787

[B28] Sodhi A Gong J Silva C Qian D Barnes PF Clinical correlates of interferon gamma production in patients with tuberculosis Clin Infect Dis 1997 25 3 617 620 10.1086/513769 9314449

[B29] Brock I Ruhwald M Lundgren B Westh H Mathiesen LR Ravn P Latent tuberculosis in HIV positive, diagnosed by the M. tuberculosis specific interferon-gamma test Respiratory research 2006 7 56 16579856 10.1186/1465-9921-7-56 PMC1523341

[B30] Luetkemeyer AF Charlebois ED Flores LL Bangsberg DR Deeks SG Martin JN Havlir DV Comparison of an interferon-gamma release assay with tuberculin skin testing in HIV-infected individuals American journal of respiratory and critical care medicine 2007 175 7 737 742 10.1164/rccm.200608-1088OC PMC1899289 17218620

[B31] Stephan C Wolf T Goetsch U Bellinger O Nisius G Oremek G Rakus Z Gottschalk R Stark S Brodt HR Comparing QuantiFERON-tuberculosis gold, T-SPOT tuberculosis and tuberculin skin test in HIV-infected individuals from a low prevalence tuberculosis country AIDS (London, England) 2008 22 18 2471 2479 19005270 10.1097/QAD.0b013e3283188415

[B32] Beffa P Zellweger A Janssens JP Wrighton-Smith P Zellweger JP Indeterminate test results of T-SPOT.TB performed under routine field conditions Eur Respir J 2008 31 4 842 846 10.1183/09031936.00117207 18057053

[B33] Doherty TM Demissie A Menzies D Andersen P Rook G Zumla A Effect of sample handling on analysis of cytokine responses to Mycobacterium tuberculosis in clinical samples using ELISA, ELISPOT and quantitative PCR J Immunol Methods 2005 298 1–2 129 141 10.1016/j.jim.2005.01.013 15847803

[B34] Kobashi Y Sugiu T Shimizu H Ohue Y Mouri K Obase Y Miyashita N Oka M Clinical evaluation of the T-SPOT.TB test for patients with indeterminate results on the QuantiFERON TB-2G test Intern Med 2009 48 3 137 142 10.2169/internalmedicine.48.1432 19182423

[B35] Adetifa IM Lugos MD Hammond A Jeffries D Donkor S Adegbola RA Hill PC Comparison of two interferon gamma release assays in the diagnosis of Mycobacterium tuberculosis infection and disease in The Gambia BMC infectious diseases 2007 7 122 17961228 10.1186/1471-2334-7-122 PMC2216027

[B36] Tsiouris SJ Coetzee D Toro PL Austin J Stein Z El-Sadr W Sensitivity analysis and potential uses of a novel gamma interferon release assay for diagnosis of tuberculosis Journal of clinical microbiology 2006 44 8 2844 2850 16891501 10.1128/JCM.02411-05 PMC1594653

[B37] Soysal A Torun T Efe S Gencer H Tahaoglu K Bakir M Evaluation of cut-off values of interferon-gamma-based assays in the diagnosis of M. tuberculosis infection Int J Tuberc Lung Dis 2008 12 1 50 56 18173877

[B38] Belgaumkar VA Gokhale NR Mahajan PM Bharadwaj R Pandit DP Deshpande S Circulating cytokine profiles in leprosy patients Leprosy review 2007 78 3 223 230 18035773

[B39] Shang X Qiu B Frait KA Hu JS Sonstein J Curtis JL Lu B Gerard C Chensue SW Chemokine receptor 1 knockout abrogates natural killer cell recruitment and impairs type-1 cytokines in lymphoid tissue during pulmonary granuloma formation The American journal of pathology 2000 157 6 2055 2063 11106578 10.1016/S0002-9440(10)64844-4 PMC1885763

[B40] Boras Z Juretic A Gagro A Pavelic L Cytokine profile of t lymphocytes from peripheral blood and bronchoalveolar lavage fluid in patients with active pulmonary tuberculosis Scand J Immunol 2007 65 3 257 264 10.1111/j.1365-3083.2006.01890.x 17309780

